# Techno‐economic analysis of membrane‐based continuous capture chromatography platforms for large‐scale antibody production

**DOI:** 10.1002/btpr.70033

**Published:** 2025-04-24

**Authors:** Juan J. Romero, Eleanor W. Jenkins, Marc R. Birtwistle, Scott M. Husson

**Affiliations:** ^1^ Department of Chemical and Biomolecular Engineering Clemson University Clemson South Carolina USA; ^2^ School of Mathematical and Statistical Sciences Clemson University Clemson South Carolina USA

**Keywords:** economic analysis, LASSO‐regression, monoclonal antibodies, multi‐objective optimization, surrogate function

## Abstract

Continuous manufacturing platforms and membrane chromatography are process technologies with the potential to reduce production costs and minimize process variability in monoclonal antibody production. This study presents a simulation and optimization framework to perform techno‐economic analyses of these strategies. Multi‐objective optimization was used to compare batch and continuous multicolumn operating modes and membrane and resin process alternatives, revealing performance differences in productivity and cost of goods attributed to variations in dynamic binding capacity, media geometry, and process residence time. From the set of optimal process configurations, we selected one membrane and one resin platform alternative yielding the highest net present values to undergo sensitivity analyses involving variations in batch cadence and product selling price. For the scenarios considered in this work, membrane continuous platforms showed benefits in the cost of goods and process mass intensity. Their shorter residence time compared to resins positions them as a viable alternative for single‐use capture chromatography. Moreover, this low residence time makes membrane platforms more flexible to changes in throughput, an essential feature for integrating capture into fully continuous processes.

## 1 INTRODUCTION

Biologics, especially monoclonal antibodies (mAbs), are used to prevent or treat many diseases and disorders. Despite their life‐saving potential, production capacity and regulatory and techno‐economic challenges^1^, ^2^ limit patient access to these drugs. Biosimilars, sharing fundamental pharmacological characteristics with reference products, have the potential to stimulate competition in the biologics market, contributing to price reductions and improving treatment accessibility.^3^ Whether for biosimilars or their reference products, high capital and operating costs pose a barrier to entry that hinders competitive growth in biologics production.^4^ Continuous manufacturing platforms address these challenges by achieving a steady‐state production process and a reduced plant footprint that can decrease capital and operating costs.^5^ Moreover, these platforms can increase product quality and consistency, facilitate adjustments in production capacity, and reduce water usage.^6^ Continuous manufacturing in the biopharmaceutical industry has been a process design goal for two decades, and substantial progress has been made in upstream and downstream technologies for implementing continuous unit operations for mAb production.^6^, ^7^

**FIGURE 1:**
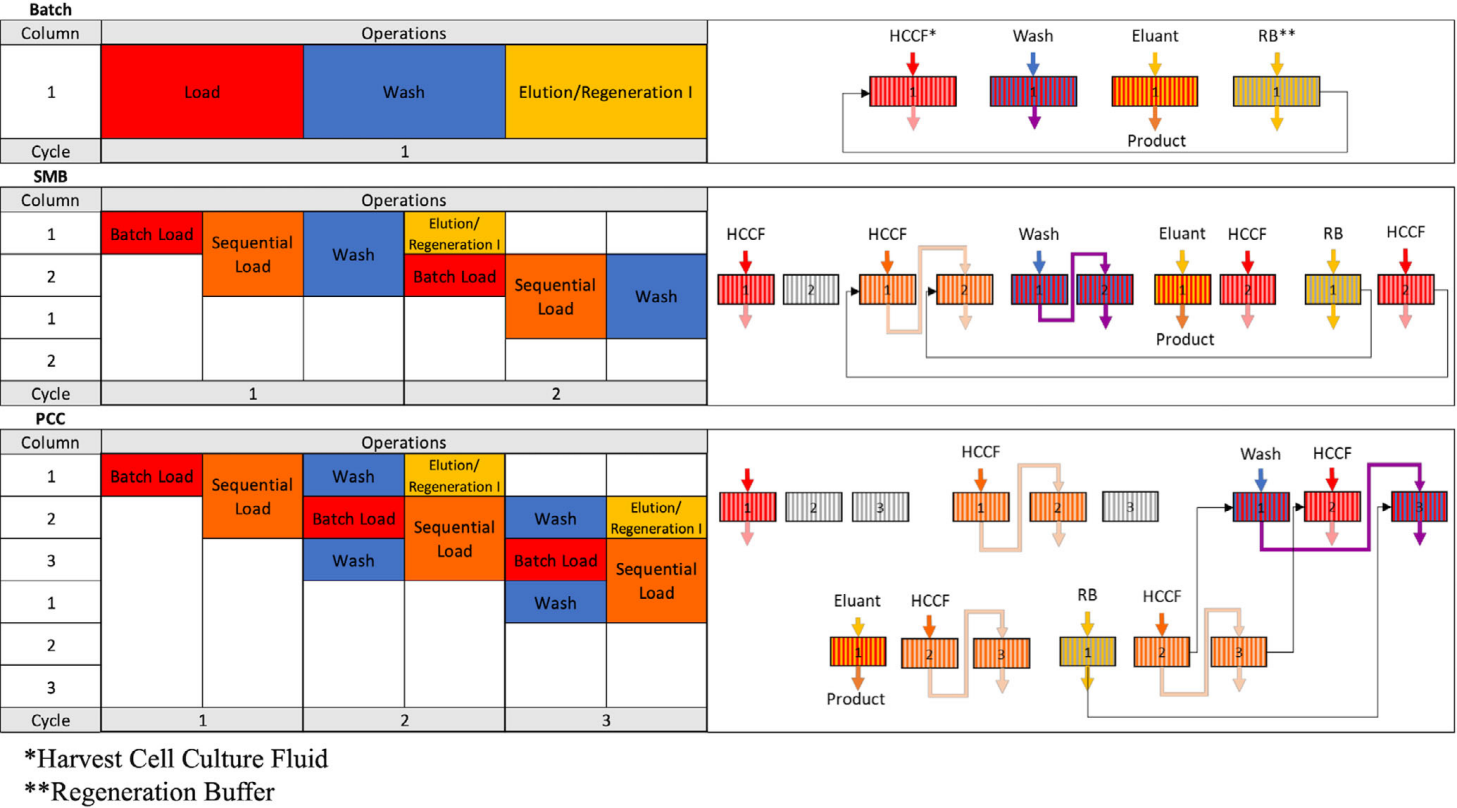
Representation of the capture operation steps for batch, SMB, and PCC platforms.

Chromatography is crucial in manufacturing mAbs and similar biologics (e.g., bispecific antibodies and antibody‐drug conjugates). This operation is used in the purification process for product capture and polishing. Capture chromatography operates in bind‐and‐elute mode. Whether in continuous, semi‐continuous, or batch operation, bind‐and‐elute chromatography follows a sequence of steps: loading, washing, elution, and regeneration. Additionally, equilibration and secondary regeneration steps are executed once per batch and are independent of the number of cycles. In multicolumn chromatography (MCC), continuous or semi‐continuous operation is achieved by loading the feed onto one column while previously loaded columns undergo washing, elution, and regeneration. After regeneration, the columns are ready to load again, and the cycle restarts. Different technologies follow this principle with varying sequences of steps.^8^ We selected simulated moving bed (SMB) chromatography and periodic countercurrent (PCC) chromatography as the MCC technologies to study because of their widespread use and commercial availability.^6^ SMB uses two or more columns operating in sequential and batch modes, providing a semi‐continuous operation. Meanwhile, PCC can achieve fully continuous operation using three or more columns. Figure 1 shows the sequence of operations in the different process configurations.

Prior studies have explored the techno‐economic feasibility of MCC platforms for clinical and commercial manufacturing.^9^, ^10^, ^11^, ^12^ Furthermore, MCC has been implemented in clinical manufacturing, delivering higher productivity and lower cost of goods (COG) than batch processes.^13^ The earlier studies were based on resin chromatography. Although resin chromatography is a proven technology, mAb adsorption to resin chromatography media is limited by a slow diffusion process that reduces throughput and productivity. In contrast, membrane chromatography is an advection‐controlled process, allowing for higher throughputs and increased productivity. Recent advancements have increased membrane binding capacities to values on par with resins, positioning membrane chromatography as a competitive technology for industrial capture processes.^14^ Despite these developments, there are no techno‐economic feasibility studies on continuous platforms using membrane capture technology, leaving a knowledge gap we address in this study.

To study the possible benefits of MCC using membrane chromatography columns, we performed a techno‐economic analysis for membrane platforms, comparing a batch process with MCC. We conducted a similar analysis for resin platforms to compare the membrane processes to these well‐established and extensively studied processes. Key performance indicators (KPIs) were selected to explore the potential benefits of the different platforms, addressing diverse organizational needs and objectives. These KPIs, defined in Table S1 of Supporting Information, use productivity as an indicator of time efficiency relative to media volume, capacity utilization (CU) as an indicator of media use efficiency, production rate as an indicator of time efficiency, COG for economic efficiency, net present value (NPV) for project economic viability, and process mass intensity (PMI) for material use efficiency.

Subset selection techniques (e.g., LASSO regression) were used to evaluate the impact of design variables (column volume (*V*_col_), number of cycles (*N*_cycles_), number of columns (*N*_col_), batch switch time (*t*_switch_)) on the KPIs, guiding the selection of optimization problem variables. These variables were used to define three bi‐objective optimization problems to determine the tradeoff between competing KPIs (productivity versus CU, COG versus production rate, and NPV versus PMI). Through this analysis, we evaluated the performance of the individual process configurations and compared the simulation results against the results of the literature on resin processes. We also identified the most profitable (i.e., highest NPV) membrane and resin process configurations and compared them in various production scenarios. We simulated the increased demand and reduced selling cost expected in biosimilar manufacturing contexts. We also compared the performance of the two technologies under conditions of single‐use capture media and fully continuous manufacturing. This approach allowed the identification of production conditions under which membranes may offer advantages over resins in terms of costs, capacity, project profitability, and plant footprint.

## 2 METHODS

### 2.1 MCC platforms

Table S2 details the process parameters used in this work. As illustrated in Figure 1, the SMB process begins by loading one column in batch mode. Subsequently, two columns are loaded in series during a sequential loading stage. Then, the columns are washed while they remain connected. Following washing, the column in position two is loaded in batch mode, while the first column undergoes elution and regeneration. The duration of this batch phase depends on the number of columns available; for instance, with only two columns, the second column is loaded in batch mode until the first is fully regenerated. In this configuration, any added column decreases the batch time by providing a new column to load sequentially while the first column is regenerated. Alternatively, it is possible to restrict the batch time and pause loading while the regeneration continues, making batch time a process variable.

PCC also operates in sequential and batch modes, but unlike SMB, loading is not interrupted during the wash step. After the sequential load, the second column is loaded in batch mode. Meanwhile, the first column is washed while connected in series with a third column. After washing, the second and third columns are loaded in series during a sequential loading stage, while the first is eluted and regenerated. The duration of the batch phase depends on the number of columns and the wash step duration, but it can be reduced by adding idle time. In our simulation, the duration of batch time is controlled by the variable *t*_switch_, which ranges from 0 to 1, indicating what fraction of the available batch time is used for loading.

### 2.2 Breakthrough simulation and surrogate function

A breakthrough curve shows the time‐dependent concentration of mAb in the chromatography column outlet stream. It is used to calculate the total mass of the product leaving the column. By comparing this mass with the mass fed to the column, we can calculate the mass of the product captured and the capture yield (quotient of mass captured and mass fed). Depending on the available data, these breakthrough curves can be obtained experimentally or, as in our case, through simulation. The models and parameters used for simulating the breakthrough curves are detailed in prior work^15^ and correspond to the Purexa™ PrA affinity membrane (Purilogics by Donaldson) and the MabSelect PrismA resin (Cytiva).

We calculated the capture yield after simulating the load and wash steps. The yield can be calculated for a batch process after a single load and wash cycle. In contrast, the yield is calculated once a steady state is achieved for MCC platforms involving multiple cycles. This condition is reached when the yield difference between the current and the previous cycle is lower than 10^−4^%.

Using this simulation, we built a library of yield values at different process conditions. Then, we created a surrogate function by interpolating the process variable(s) among the evaluated conditions. Product load (grams of mAb/media volume) was the only process variable for batch processes. Switch time was added for SMB and PCC platforms. We used a two‐dimensional interpolation method for the surrogate function in these instances. Piecewise linear interpolations were performed in MATLAB using the built‐in interpolation functions (interp1 and interp2). Preliminary trials found linear interpolation to be as effective as higher‐order methods. The number of columns impacts the total process time but not the breakthrough curve; thus, it was excluded as a variable for the dynamic simulation. The surrogate function was validated by comparing the yield values derived from interpolation with those obtained through dynamic breakthrough curve simulation using the Root Mean Squared Error (RMSE). The validation process used randomly generated conditions within the search space of the proposed production platforms to generate the simulated and interpolated yield values.

### 2.3 Cost model and production scenario

The process alternatives explored in this study focus on modifications to the capture chromatography unit operation. Instead of simulating the entire process, we divided it into distinct blocks: an initial block for the upstream process (USP), a capture process simulation, and a block for the remaining operations in the downstream process (DSP) (Figure S3). The SuperPro built‐in mAb example served as the foundation for our flow sheet simulation. From this example, we gathered information on the USP and DSP blocks, including capital and operational expenditures (Capex and Opex), yield, and production scale. Table S4 summarizes the main cost and production parameters. The selling price of the product was reduced from 200 USD/g to 40 USD/g according to the estimated 30% reduction for biosimilars compared to the reference product in the US, Japan, and European markets.^2^

### 2.4 KPI definition

Table S1 shows the definitions of the selected KPI. We chose yield, CU, and productivity because of their widespread use in chromatography feasibility analyses.^16^, ^17^, ^18^ Yield is a crucial measure of process performance, focusing on product recovery, which is the primary aim of the capture operation. CU quantifies the efficiency of media volume usage, a significant contributor to operating costs. Productivity allows us to balance the mass of product obtained against the process duration and media volume used, providing a valuable metric to compare process performance across different production scales. We included production rate as a KPI specific to processing time.

While these indicators offer insights into the potential economic efficiency of the operation, our cost model incorporates specific economic indicators like the COG to compare capture‐operating costs normalized by the mass of mAb produced. Despite its usefulness and widespread use, COG solely considers Opex and ignores Capex. To address this, we introduced NPV to compare various process alternatives within the context of the economic analysis of investments. Finally, following previous literature, we chose PMI to assess plant resource efficiency, influencing process sustainability and footprint.^9^

### 2.5 Variable selection

In capture chromatography, various parameters can be adjusted, including media parameters such as binding capacity, membrane thickness, or porosity; module design parameters like media volume, the number of columns, or maximum flow rate; and operating parameters like the number of cycles per batch, residence time, or step duration. This study aimed to explore the implementation of membrane technology. Consequently, we opted for commercial chromatography media in commonly available module configurations (pre‐packed columns and cassettes). Each cassette contains a membrane layer through which the feed flows. Several modules can be stacked parallel in a cassette holder to achieve the required media volume. We selected *V*_col_ as a variable due to the significant impact of this consumable on operating costs.^19^ *N*_cycles_ was chosen because of its influence on process time and yield.

In batch platforms, *V*_col_ and *N*_cycles_ are the sole process variables. For PCC and SMB platforms, the *N*_col_ and *t*_switch_ parameters have been added. To optimize all process alternatives efficiently, we identified the variables that significantly affect process performance. To achieve this, we applied LASSO regression to a set of sample points within the search space, exploring the influence of different variables on the six KPIs. LASSO regression produces coefficients for a multivariate linear regression by minimizing the squared error plus a one‐norm penalty term.^20^ The error function incorporates a lambda parameter that diminishes the impact of process variables on the model for a KPI. As the lambda value increases, the coefficients of the LASSO regression trend toward zero, starting with variables that have a lower influence on the KPI.

### 2.6 Multi‐objective optimization (MOO) problem formulation

We explored the tradeoff between competing objectives by formulating bi‐objective optimization problems for all platforms. Specifically, we considered the following objectives: CU versus productivity to compare our processes with the literature; COG versus production rate to compare cost and speed; and NPV versus PMI to compare economic gain and resource impact. The multi‐objective genetic algorithm function in MATLAB 2022 (*gamultiobj*) was employed for mixed‐integer optimization (Global Optimization Toolbox, www.mathworks.com). Parameters were configured with a maximum population size of 200 and a maximum of five generations. The standard form of the problem is presented in Equations 1‐5, with f and g being any objective function. In this general representation, four variables are considered, although this number may vary depending on the specific objective functions.

(1) $$\min_{x}f\left(x\right),g\left(x\right)$$

With

(2) $$x=\left[N_{\text{cycles}},N_{\mathrm{col}},V_{\mathrm{col}},t_{\text{switch}}\right]$$

St.:

(3) LL≤x≤UL

(4) $$\frac{\text{Vbatch}÷\left(N_{\text{cycles}}*N_{\mathrm{col}}\right)}{\text{Vcol}}\leq 50$$

(5) $$\frac{\text{Vcol}÷N_{\mathrm{col}}}{\mathrm{RT}}\leq 200L/h$$

We set *N*_col_, *N*_cycles_, and *V*_col_ as integer variables and *t*_switch_ as a continuous variable. The number of columns was constrained to be an integer between 2 and 10 for SMB and 3 and 10 for PCC. *N*_cycles_ was set between 1 and 25. *V*_col_ was selected as an integer between 1 and 100 for membranes, corresponding to the number of 1.6 L cassettes in the process skid. For resins, volumes correspond to columns with a bed height of 20 cm for batch and 10 cm for MCC configurations and diameters from 20 to 200 cm at 20 cm intervals. Lastly, t_switch_ was in the interval [0, 1], with zero representing no batch time and one being the maximum batch time for the corresponding MCC mode. In addition to setting upper and lower limits (UL and LL) for the process variables, the largest product load was constrained (up to 250 g of mAb/L of media) to prevent extrapolation beyond the surrogate function library. Another constraint limits the maximum flow rate to the skid specifications (200 L/h).

## 3 RESULTS AND DISCUSSION

### 3.1 Surrogate function validation

As the simulation complexity increases, so does its computing time, which becomes impractical for users needing access to high‐performance computing. This limits the applicability of the simulation framework for process design in industrial settings. Using a surrogate function instead of simulating a system of differential equations reduces computing time significantly. In a prior study,^21^ we achieved a 93% decrease in computing time for batch capture chromatography simulations. The reduction in computing time for MCC configurations is even more significant. MCC platforms must achieve a steady state by simulating multiple load and wash cycles, resulting in longer simulations. For resins (solved using a finite volume method for a shrinking core model simulation^17^), the average computing time for an MCC simulation was 32.5 ± 18.1 min. For membranes (solved using a finite element method for an instantaneous adsorption model simulation^22^), the simulation computing time was 105.2 ± 39.0 min. The interpolation‐based surrogate function reduced computing times by 99.988% and 99.996%, respectively, with an average simulation time of 13 s. However, reducing computing time comes with a trade‐off of decreasing the degrees of freedom in the simulation, requiring fixed residence time (RT) and media height values.

The accuracy of the surrogate function was validated by comparing the product yield outcomes for 20 sets of process conditions chosen from the search space for each process. These parameters were used in a finite element simulation and compared with the results of the surrogate function. Figure S5 shows these results and the surrogate representations of yield versus product load for six capture chromatography processes. The error was equal to or less than the required accuracy threshold of 1.0 × 10^−3^ for most points. This threshold was selected to match the precision of the original experimental data used in the model parameter fitting. Although some residual values fell between 1 × 10^−3^ and 1.2 × 10^−3^, the RMSE for all six data sets remained below the threshold (see details in the figure legend), showing that the surrogate function was accurate for simulating the processes.

### 3.2 LASSO regression analysis

Using the surrogate function simulation allowed us to generate extensive datasets and assess the impact of the process variables on the KPIs. We used it to evaluate 193 points for batch platforms and 1906 points for MCC platforms under random conditions within their respective search spaces. These point quantities represent 10% and 1% of the membrane search space for membrane batch and SMB configurations (after discretizing switch time in 0.1 intervals). These platforms feature the most extensive search space for batch and MCC configurations. Subsequently, we applied LASSO regression to these datasets, from which we analyzed lambda values to understand which process variables were most important for the different KPIs (Table S6). This approach eliminates unnecessary variables in the optimization problem formulation, reducing computing times and enhancing accuracy.

Overall, we observed a low impact of *t*_switch_ in all KPIs, while *V*_col_ considerably influences all KPIs. Furthermore, variables related to product load are always significant. This analysis showed how *t*_switch_ was not influential for any MCC membrane KPI. It was not influential for 79% of the MCC resin KPI. This allowed us to eliminate one variable in 9 out of the 12 optimization problems for MCC platforms (Table S7). Employing three variables instead of four reduced the number of simulations from 2000 to 1000, thereby decreasing computing time by half.

### 3.3 Optimization

**FIGURE 2:**
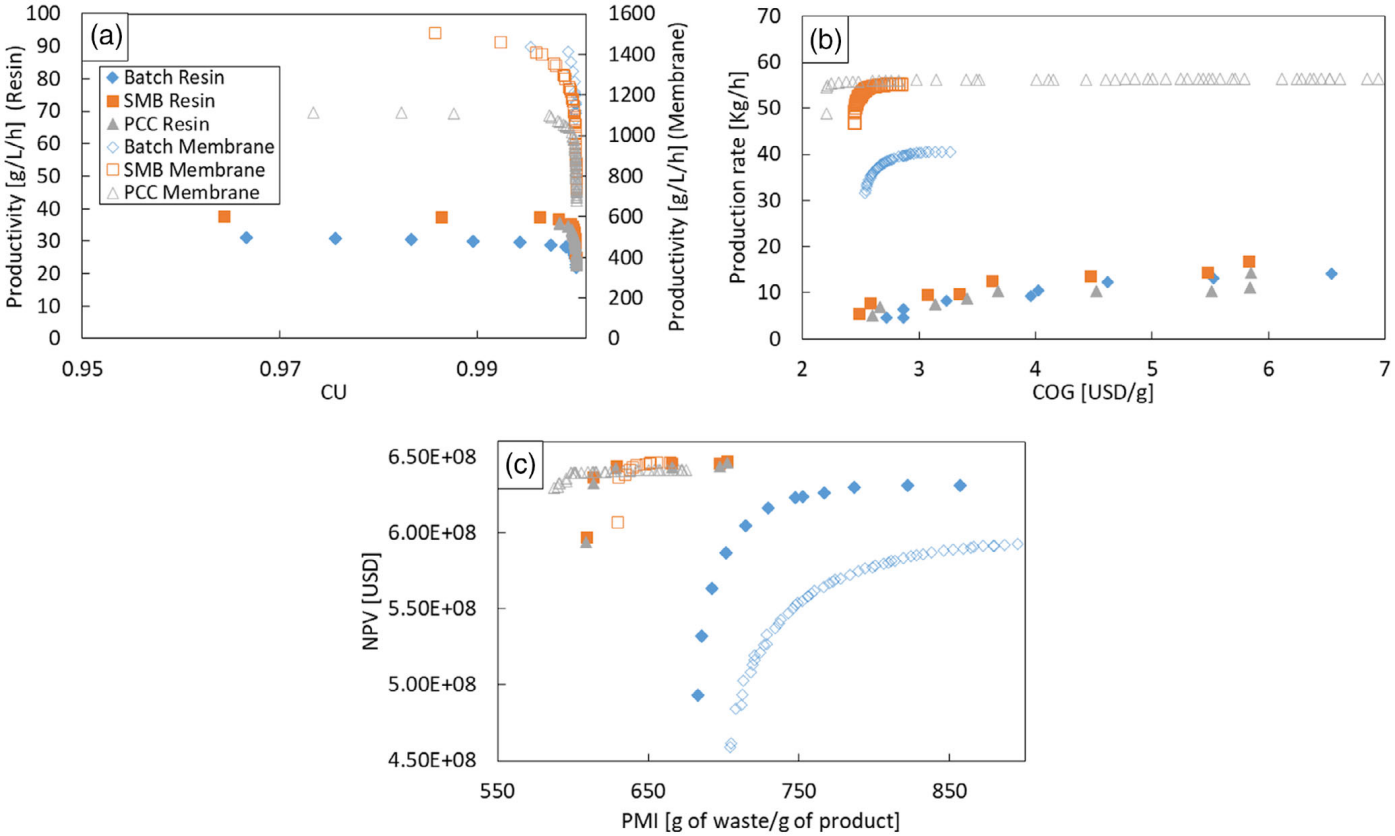
Pareto fronts for (a) productivity versus CU‐note the different productivity scales for resins and membrane platforms; (b) production rate versus COG, illustrating how membrane platforms offer lower COG and higher production rate than their resin counterparts; and (c) NPV versus PMI, illustrating how resin and membrane SMB platforms have the highest NPV, while the PCC membrane platforms have the lowest PMI.

The three bi‐objective optimization problems were solved for each of the six process alternatives, with the results presented in Figure 2. Figure 2a shows the Pareto fronts for productivity versus CU. Resin platforms behave consistently with the literature, showing improvements in both indicators for PCC and SMB compared to the batch process.^17^, ^23^ MCC configurations allow greater product breakthrough on the first column during loading without losing product, thereby enhancing both CU and productivity. Additionally, the MCC configurations reduce total process time by running elution and regeneration parallel to loading, thereby increasing productivity.

However, in the case of membranes, a deviation from this behavior is observed due to differences in the sequential load step. For resins, the sequential loading of two small columns behaves similarly to a single column with equivalent volume because the cross‐sectional area can be kept constant while changing column height. Using two columns with constant membrane thickness reduces the cross‐sectional area, affects the feed flow rate, and decreases productivity.

When comparing SMB and PCC, we see that SMB reaches higher productivity for the same CU under the conditions of the problem. PCC configurations are slower than SMB for the same product load despite being fully continuous. Having more columns in PCC increases the number of cycles with smaller and faster loads. Consequently, regeneration time becomes a bottleneck, and idle time must be added after the sequential load operation (Figure 1). In contrast, SMB sequential load time can be reduced without incurring idle times because no other process runs simultaneously. This compensates for the semi‐continuous operation.

Idle time could be avoided in PCC by increasing the number of columns while reducing the number of cycles to keep the same product load. However, having spare columns ready for the sequential load means having a lower media volume for this step, resulting in lower yield and CU. A lower media volume for the sequential and batch loading steps also necessitates lower flow rates to keep the same RT, resulting in lower production rates. As a result, optimal solutions for SMB and PCC consistently were obtained with two and three columns, respectively.

CU is used as an indirect indicator of economic benefit due to the significant cost of media.^19^ The economic model in our simulation framework directly calculates COG. Thus, we examined the relationship between COG and production rate. The COG values obtained across the different process configurations are consistent with similar studies, such as those by Bansode et al., who report capture chromatography COG values of approximately 7 USD/g for batch and 3 USD/g for continuous protein A capture.^19^ In Figure 2b, we observed a distinct behavior in production rate compared to productivity. While productivity aims for efficient CU, the production rate prioritizes the fastest process, typically using the largest media volume to ensure high throughput. However, the flow rate can only be increased to the capacity of the skid pumps (200 L/h). This constraint narrows the gap in the production rate between membrane and resin platforms. The flow rate is also limited by minimum residence time requirements, which were kept constant in our model. Additionally, factors such as column pressure and linear velocity could impose further limitations, though these were beyond the scope of this study. The membrane platforms show more optimal points, aligning with the larger search space produced by the cassette module configuration. This enables fine‐tuning process conditions to achieve the desired tradeoff with greater precision according to user preferences.

The PCC membrane configuration yields the best performance in both KPIs. The shorter RTs of membrane processes result in higher production rates and lower labor costs. Furthermore, the fully continuous operation provided by PCC configurations reduces column volume by increasing the number of cycles without extending the total process time. The lower media volume also reduces buffer requirements for regeneration II and equilibration operations.

Finally, we explored the economic and environmental impacts of the processes by analyzing the tradeoff between NPV and PMI. PMI aims to minimize waste generation, which favors small media volumes and high CU. NPV looks to reduce the COG and maximize yield, which requires a balanced product load. Product loss occurs if the product load is too high, and media is underutilized if it is too low. This tradeoff is reflected in the Pareto fronts depicted in Figure 2c.

**FIGURE 3:**
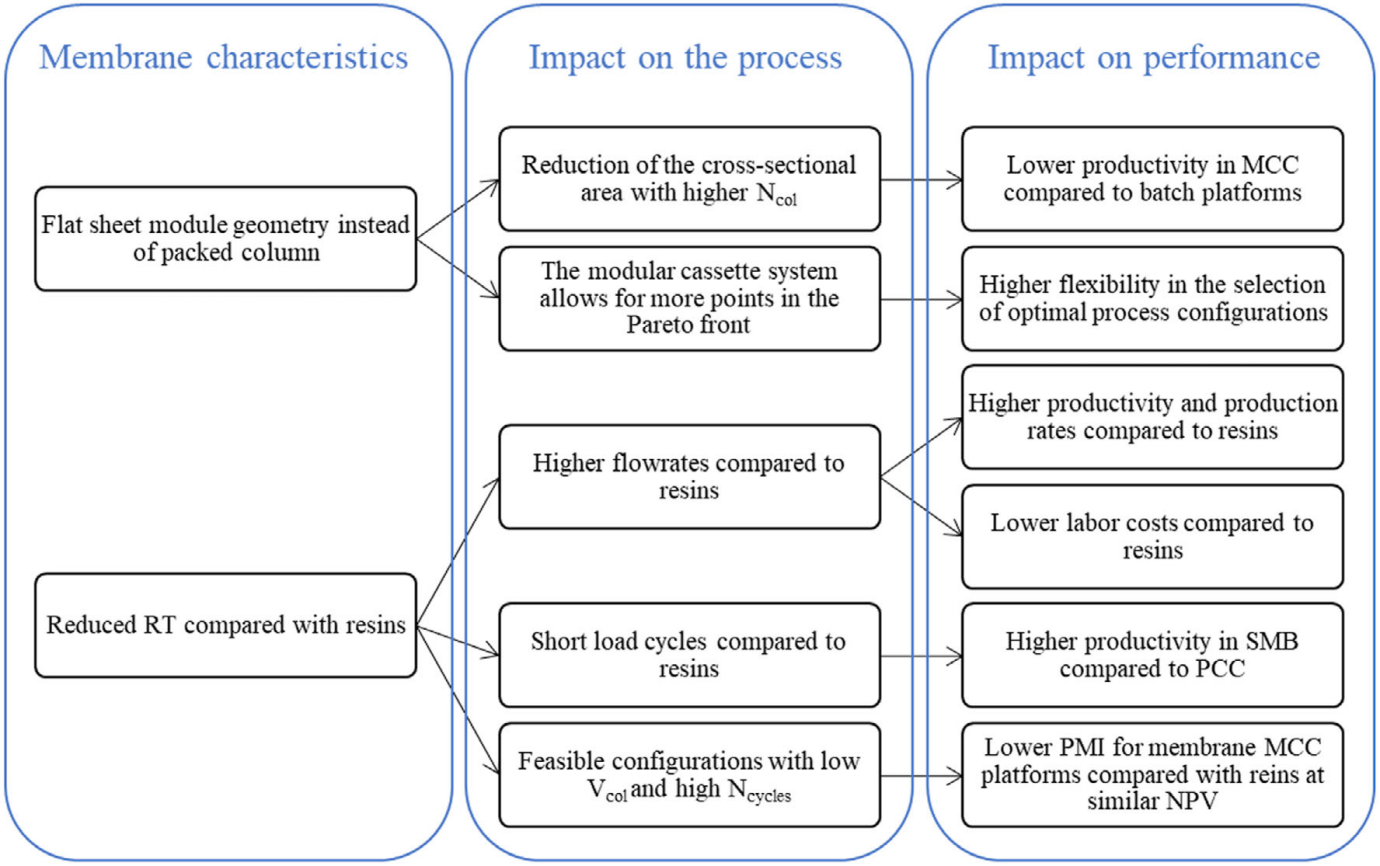
Summary of observations particular to membrane platforms.

Due to low media volume, the PCC membrane platforms have the lowest PMI values. The highest NPV values are observed in the SMB configurations due to the higher yield in two‐column configurations in which all the media volume is available for the sequential load. Since affinity membrane modules for large‐scale capture are not yet available, buffer consumption values per unit of media volume for membrane platforms are assumed to be the same as for resins. However, differences in module dead volumes and flow requirements could alter buffer usage, impacting both PMI and NPV. Figure 3 summarizes the main differences between membrane and resin platforms and their impacts.

### 3.4 Sensitivity analyses on production conditions

In this section, we evaluate how production conditions, rather than process variables, influence the performance of membrane and resin platforms. Our analysis focuses on economic performance, selecting the configurations that achieved the highest NPV. The optimal operating conditions for this KPI, identified in the previous section, serve as the basis for this assessment. First, we present economic evaluation results for the optimal SMB membrane and resin processes. Thereafter, we present results from the sensitivity analyses we conducted under varying production conditions. The sensitivity analyses first focused on the typical conditions for biosimilar products, characterized by high production volumes and lower selling prices. Next, we examined single‐use capture platforms, an appealing alternative that minimizes the burden of strict cleaning protocols required to ensure the sterility of the final product.^24^ Finally, we evaluated the performance differences between the two technologies within fully continuous manufacturing operations.

#### 3.4.1 Optimal SMB membrane and resin products with an assumed selling price

Table 1 presents the economic details of the SMB resin and membrane process configurations, yielding the highest NPV for the case with a $140/g mAb selling price. Overall, the performance of the two platforms is highly similar in this production scenario. The resin platform has higher operating costs, that is, higher COG. However, it has a higher yield that translates into approximately 3 kg more product per year, which offsets the higher operating costs. The higher capacity of the optimized capture process configuration drives this yield increase. In the breakdown of operating costs, we observe how a slightly higher dynamic binding capacity of resins leads to lower media utilization. However, the extended process time increases labor costs, and the larger column volume raises buffer use and associated costs.

**TABLE 1:** Economic evaluation results for optimal SMB membrane and resin processes.

Item	Units	Membrane	Resin
*Capture*		
*V*_col_	L	44.8	226.2
*N*_cycles_		21	4
*N*_col_		2	2
Capture yield		0.9982	0.9998
Capture time	h	1.28	9.35
Capture flow through	L/min	188	26
Capture productivity	g/h/L	1179	32
Capture CU		0.870	0.836
Media utilization	L/year	300	283
Buffer consumption	L/g of product	0.377	0.415
PMI	g of waste/g of product	665	703
*Costs*			
Raw materials	USD/year	2,439,000	2,690,000
Labor‐Dependent	USD/year	58,000	442,000
Facility‐Dependent	USD/year	197,000	197,000
Laboratory/QC/QA	USD/year	9000	66,000
Chromatography media	USD/year	5,786,315	5,564,769
Other consumables	USD/year	230,300	230,300
Waste Treatment/Disposal	USD/year	70,000	75,000
Total capture operating cost	USD/year	8,789,615	9,265,069
Capture COG	USD/g	2.76	2.90
*Economic Evaluation*		
Total USP operating cost	USD/year	60,217,340	60,217,340
Total DSP operating cost*	USD/year	17,426,378	17,426,378
Total COG	USD/g	36.30	36.44
Total operating cost	USD/year	86,433,333	86,908,787
Total capital investment	USD/year	571,270,000	571,270,000
DSP yield		0.7078	0.7090
Batch capacity	g/batch	50,668	50,750
Batches per year		47	47
Product throughput	g/year	2,381,418	2,385,260
Selling price	USD/g of product	140	140
Revenues	USD/year	333,399,000	333,936,000
Gross margin	%	74.07	73.97
Return on investment	%	25.94	25.94
Payback time	years	3.86	3.85
IRR (after taxes)	%	25.88	25.88
NPV (at 7.0% interest)	$	646,609,000	646,702,000

* Excluding capture operating costs.

#### 3.4.2 Impact of demand and price of final product

**FIGURE 4:**
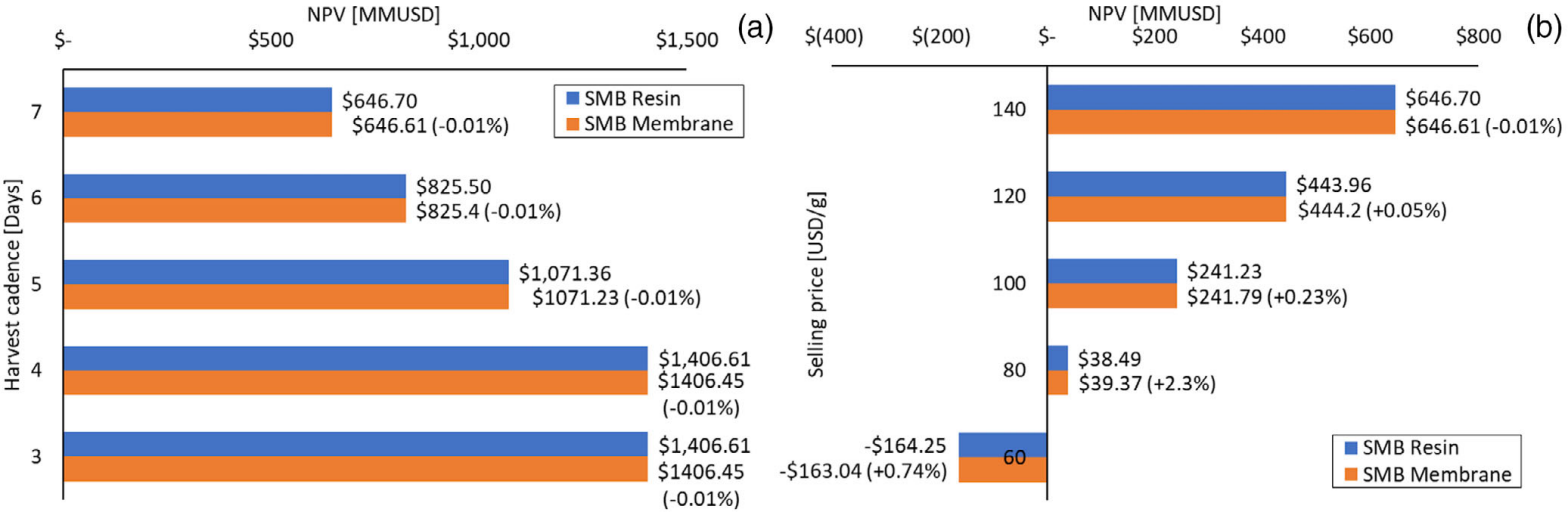
Sensitivity analyses of increased harvest cadence (a) and decreased mAb selling price (b) for optimal SMB membrane and resin processes.

The similar performance of the two platforms for a $140/g mAb selling price remains when we intensify production by reducing the harvest cadence time, as shown in Figure 4a. In these scenarios, the NPV increases as the number of batches per year reaches the maximum of 81 batches. Beyond this point (4‐day harvest cadence), there is no further improvement in NPV associated with a faster harvest cadence.

Conversely, when the selling price of the product is reduced, we see how the benefit of lower COG positions the membrane platform as more profitable (higher NPV). Some sources estimate mAb market prices at approximately $20,000 USD/g,^7^, ^19^ with lower‐end values around $4000 USD/g.^25^ Simulating the process with a $4000 USD/g product selling price resulted in NPV values of 39,712 and 39,776 million USD for the SMB membrane and resin platforms, respectively. This is consistent with estimates reported by Grilo et al. for a process using the same pricing assumption.^26^ However, market prices often exceed the internal transfer price assigned to a manufacturing facility as a business unit. Given this uncertainty, we adopted the default SuperPro value for product revenue.

Despite having marginal differences in terms of NPV for all cases considered, the membrane process is expected to offer other economic benefits. Although not considered in our cost model, a lower PMI associated with membrane processes is linked to smaller auxiliary facilities for wastewater treatment and buffer production. The considerably smaller media volume also reduces initial consumable costs and the overall equipment cost. The initial capital investment is reduced, lowering the barrier of entry for new competitors in the biosimilar market. Additionally, the membrane platform offers greater flexibility in batch size due to its higher number of cycles. The ability to adjust the installed capacity in response to changing demands decreases the risk associated with the investment.

#### 3.4.3 Single‐use platforms

The fast membrane throughput poses an additional advantage in the context of single‐use platforms. For chromatography, the high cost of the stationary media makes large disposable modules an unfeasible alternative. However, the volume of stationary media required depends on the load per cycle and can be reduced by increasing the number of cycles.^27^ For economic viability, the media should be used for its entire lifetime (set to 150 cycles in our case). Increasing the number of capture cycles implies reducing the column volume to maintain the desired product load. These configurations with smaller and more numerous cycles have longer total process times.

By requiring a resin column to be used for its full lifetime of 150 cycles, the SMB resin capture platform becomes a bottleneck for the entire DSP. In this configuration, the maximum number of batches per year decreases from 47 in the optimal configuration presented in Section 3.4.1 to 25, reducing the NPV to 114 MMUSD (an 82% reduction compared with the optimal configuration). In contrast, using the membrane platform in a 150‐cycle configuration reduces NPV by a mere 0.17%. This slight decrease in profitability is easily offset by the potential advantages of single‐use technologies, including reduced contamination risks, lower capital investment, and decreased material and labor costs.^24^, ^28^, ^29^

#### 3.4.4 Fully continuous operation

**FIGURE 5:**
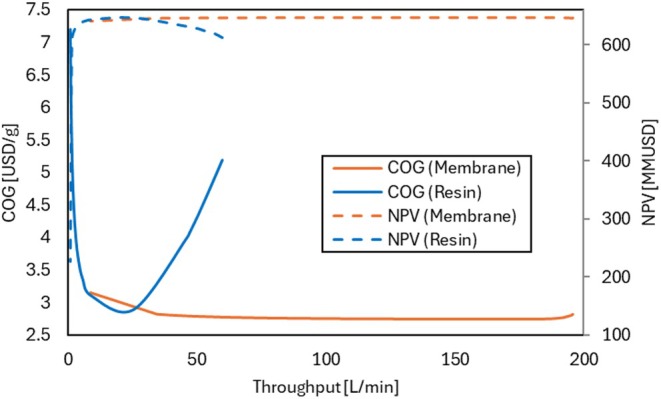
NPV and COG for SMB membrane and resin platforms as functions of capture throughput.

In the previous scenarios, only process improvements to the capture operation were considered, leaving other parts of the process unchanged. However, MCC operations should be implemented with continuous bioreactor technologies to reap the benefits of continuous or semi‐continuous production. In such processes, the capture feed is a constant stream instead of a bulk batch. The capture throughput must match this stream flow rate to process this feed continuously. To maintain an optimal yield, the product load is kept fixed, leaving throughput as a function of column volume. Within their column, volume ranges (6.28–628 L for resins and 1.6–160 L for membranes), the resin platform can process feed flow rates between 0.80 and 60 L/min, while the membrane platform can process feed flow rates between 8.8 and 196 L/min. Figure 5 explores the economic performance of the two platforms across these throughput ranges.

The membrane platform offers stable NPV and COG across a wide range of throughputs, with a minor decrease in performance only when approaching 200 L/h (the skid pump maximum capacity). Beyond this point, reducing the number of cycles does not reduce process time and labor costs. However, increased media volume impacts buffer consumption, thereby increasing material costs. While the competing influence of labor and material costs also affects the resin platform, the dramatic performance losses observed at low throughputs are caused by the reduction in total product output. To maintain the desired production rate, every operation must have a minimum throughput of 1.4 L/min. When capture throughput falls below this threshold, it becomes a bottleneck, limiting the amount of product that can be processed annually.

Overall, MCC membrane platforms enable high process throughput and offer a flexible range of operating conditions. While these characteristics may not be advantageous over resin platforms in a traditional batch process, they provide important benefits for single‐use and fully continuous platforms. Additionally, the rapid cycling capability of membranes, regardless of the scenario, offers the added advantage of low residence times in the capture process. It is valuable for processing delicate molecules, where long times on the column can lead to product degradation.

## 4 CONCLUSIONS

This study of key performance indicators for mAb production demonstrates how modeling and optimization tools can inform decisions on process alternatives. The results of these optimization studies highlighted the distinct behavior of membrane platforms in MCC operating mode compared with resin platforms. As expected, membrane platforms had higher production rates than resin platforms and were limited only by the capacity of the skid pumps. Regarding COG, we observed that the optimal points for membrane processes were at lower media volumes than resin processes. This is attributed to the higher throughput of membrane platforms, making the increase in cycles economically viable. Consequently, membrane processes have lower process PMI and COG.

Our sensitivity analysis of scenario conditions revealed that, in large‐scale mAb production, SMB resin and membrane platforms perform similarly for a mAb selling price of $140/g, as measured by NPV. As product value decreases, particularly as expected with biosimilars, membrane processes with lower operational costs are marginally more profitable alternatives. In scenarios with single‐use capture platforms and fully continuous production, membrane processes offer a more distinct advantage over resins. Membranes with lower RT than resins enable process configurations with smaller columns that use more numerous cycles, an important feature for single‐use platforms. Membranes also support a much broader range of economically feasible throughputs, making them a more attractive option when considering the integration of MCC into a fully continuous production process.

Future studies can further explore the implications of these technologies by expanding the simulation framework to account for equipment resizing downstream of the capture operation based on elution volumes. As new membrane modules become available, updating study parameters to reflect buffer utilization differences due to housing design will provide a more accurate assessment of membrane‐based processes.

## AUTHOR CONTRIBUTIONS

**Juan J Romero:** Conceptualization (supporting); data curation (lead); formal analysis (lead); methodology (equal); software (lead); visualization (lead); writing – original draft (lead); writing – review and editing (equal). **Eleanor W. Jenkins:** Conceptualization (supporting); formal analysis (supporting); methodology (equal); software (supporting); visualization (supporting); project administration (supporting); supervision (equal); writing – review and editing (equal). **Marc R. Birtwistle:** Conceptualization (supporting); formal analysis (supporting); methodology (equal); visualization (supporting); writing – review and editing (equal). **Scott M. Husson:** Conceptualization (lead); formal analysis (supporting); methodology (equal); visualization (supporting); project administration (lead); supervision (equal); funding acquisition (lead); writing – review and editing (equal).

## FUNDING INFORMATION

This research was funded by the National Institute of General Medical Sciences of the National Institutes of Health, award numbers R15GM131341 and R35GM141891, and the National Science Foundation Division of Mathematical Sciences under award DMS‐2011902.

## CONFLICT OF INTEREST STATEMENT

Scott Husson has an ongoing financial interest in Purilogics and provides consulting services to the Company. The funders had no role in the design of the study, in the collection, analysis, or interpretation of data, in the writing of the manuscript, or in the decision to publish the results.

## Supporting information


**Data S1.**.

## Data Availability

The data that support the findings of this study are available from the corresponding author upon reasonable request.
