# Supplementary material for: Techno‐economic analysis of membrane‐based continuous capture chromatography platforms for large‐scale antibody production
Source: Biotechnol Prog. 2025 Apr 24;41(5):e70033. doi: 10.1002/btpr.70033 (PMC12353975; doi:10.1002/btpr.70033)
Supplement: Supplementary file 1 — Data S1.. [file BTPR-41-e70033-s001.docx]

**Supplementary material**

**Techno-economic analysis of membrane-based continuous capture chromatography platforms for large-scale antibody production**

Juan J. Romero^1^, Eleanor W. Jenkins^2^, Marc R. Birtwistle^1^, Scott M. Husson^1,*^

^1^ Department of Chemical and Biomolecular Engineering, Clemson University, Clemson, SC 29634 USA

^2^ School of Mathematical and Statistical Sciences, Clemson University, Clemson, SC 29634 USA

*Corresponding author: [shusson@clemson.edu](mailto:shusson@clemson.edu)

**Contents**

**Table S1.** Definitions of the KPI used as objective functions.

**Table S2.** Fixed parameters for capture chromatography.

**Figure S3.** SuperPro Designer flowsheet for the simulated process.

**Table S4.** Cost model and production scenario parameters.

**Figure S5.** Surrogate functions and validation points for describing yield versus relative load for six process platforms

**Table S6.** Normalized minimum lambda value for zero-valued coefficients in LASSO regression for different platforms and KPI.

**Table S7.** Variables used for the formulation of the bi-objective optimization problem.

**Table S8.** General impact of independent variable increment on KPI.

**Table S1.** Definitions of the KPI used as objective functions.

| **Indicator** | **Definition** | **Variables** | |
| --- | --- | --- | --- |
| Yield | $\frac{Total mass of mAb recovered in elution}{Total mass of mAb fed in load}$ | Total mass of mAb recovered in elution | Function of media volume (V_col_), DBC, total mass of mAb fed in load, and elution yield |
|  |  | Total mass of mAb fed in load | Function of the amount of material to be processed (batch size) and the number of cycles (N_cycles_) in which it is divided. |
| Capacity utilization (CU) | $\frac{Total mass of mAb adsorbed before elution}{EBC*Media volume}$ | Total mass of mAb adsorbed before elution | Function of the column volume (V_col_), DBC and how much material is loaded. |
| Costs of Goods (COG) | $\frac{Total batch operation cost}{Total mass of mAb recovered in elution}$ | Total batch operation cost | Function of the cost of consumables, buffer, labor, utilities and waste treatment associated with producing a product batch. |
| Productivity | $\frac{Total mass of mAb recovered in elution}{Batch time*Media Volume}$ | Batch time | Function of all the parameters listed in table S2. In MCC operation, switch time (t_switch_) also affects this variable. |
| Production rate | $\frac{Total mass of mAb recovered in elution}{Batch time}$ |  |  |
| Net present value  (NPV) | $\sum_{i=0}^{n} \frac{Net cash flow in year i}{{(1+internal rate of return)}^{i}}$ | Net cash flow in year i | Function of the selling price of the product, the number of batches per year, batch size, global yield, total batch operation cost, equipment depreciation, and facility construction cost. |
| Process Mass Intensity (PMI) | $\frac{Total mass of materials used in the batch}{Total mass of mAb recovered in elution}$ | Total mass of materials used in the batch | Function of initial batch concentration, V_col_, and the membrane volumes for washing, elution, regeneration, and equilibration. |

Note: Some indicators share definition variables.

**Table S2.** Fixed parameters for capture chromatography.

| **Parameter** | **Value** |
| --- | --- |
| Ratio V_membrane_/V_void_ | 0.55 |
| Resin column residence time (RT) | 180 s |
| Membrane column RT | 5 s |
| Resin bed height | 20 cm |
| Membrane thickness | 0.04 cm |
| Wash volume (membrane volumes, MV) | 10 |
| Wash flow velocity (v) | 225 cm/h |
| Elution volume (MV) | 4 |
| Elution v | 150 cm/h |
| Regeneration I volume (MV) | 6 |
| Regeneration I v | 300 cm/h |
| Regeneration II volume (MV) | 9 |
| Regeneration II v | 300 cm/h |
| Equilibration volume (MV) | 6 |
| Equilibration v | 300 cm/h |
| Maximum volumetric flow rate | 200 L/h |


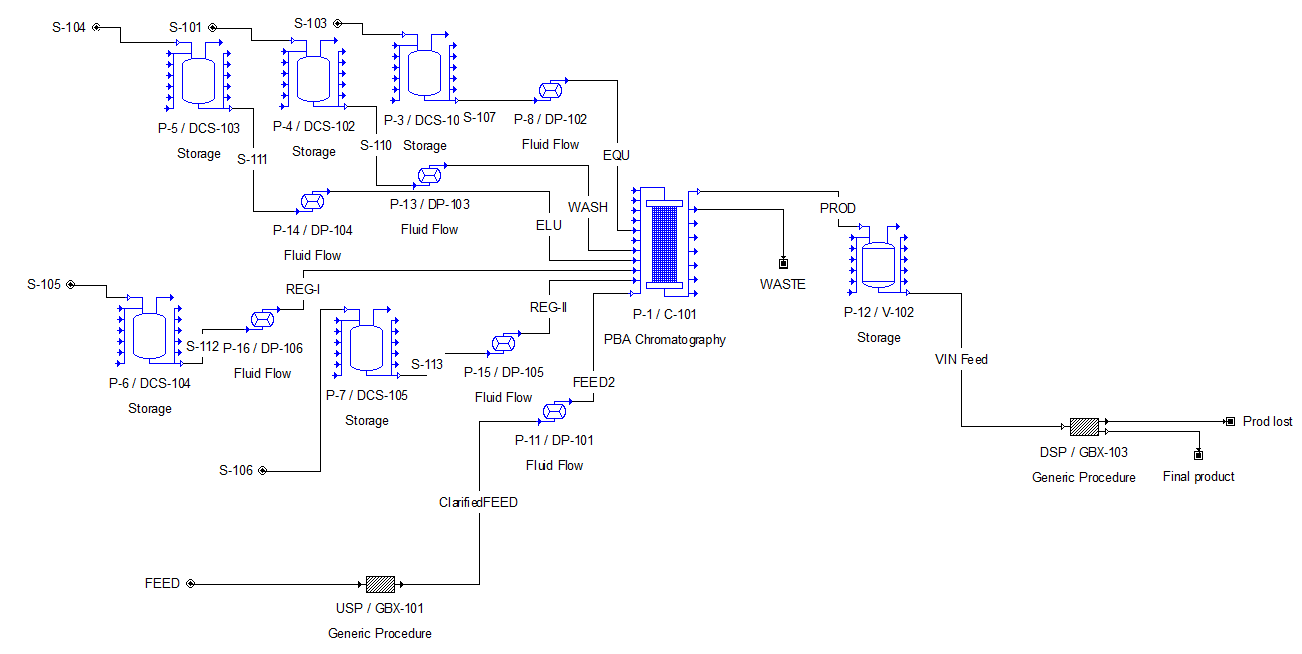


**Figure S3.** SuperPro Designer flowsheet for the simulated process. The process solutions contained in disposable containers for storage (DCS) are fed with diaphragm pumps (DP) into the chromatography unit (C). The product is recovered in a vessel (V).

**Table S4.** Cost model and production scenario parameters.

| **Parameter** | **Value** | **References** |
| --- | --- | --- |
| Batches/yr | 81 | SuperPro, www.intelligen.com |
| V_batch_ | 14,430 L | SuperPro, www.intelligen.com |
| Feed mAb titer | 5 mg/mL |  |
| Media replacement frequency | 150 Cycles | Scaling Fibro chromatography, www.cytivalifesciences.com |
| Harvest cadence | 7 days |  |
| Labor schedule | 24/7 |  |
| Operators per shift | 6 |  |
| Labor FTE cost | $75/FTE hour |  |
| Supervisory labor | 15% of FTE hours | (Grilo et al., 2017) |
| Lab quality control | 15% of FTE hours | (Grilo et al., 2017) |
| Utilities | $26,100/day | SuperPro, www.intelligen.com |
| Waste treatment | $0.05/L | SuperPro, www.intelligen.com |
| Media cost | $21,542/L | MabSelect PrismA^TM^, ww.cytivalifesciences.com |
| Buffer cost | $3/L | SuperPro, www.intelligen.com |
| Total CapEx | $570,261,878 | SuperPro, www.intelligen.com |
| USP OpEx | $1,281,220/batch | SuperPro, www.intelligen.com |
| DSP OpEx | $370,774/batch | SuperPro, www.intelligen.com |
| Selling price | $140/g of product | SuperPro, www.intelligen.com |


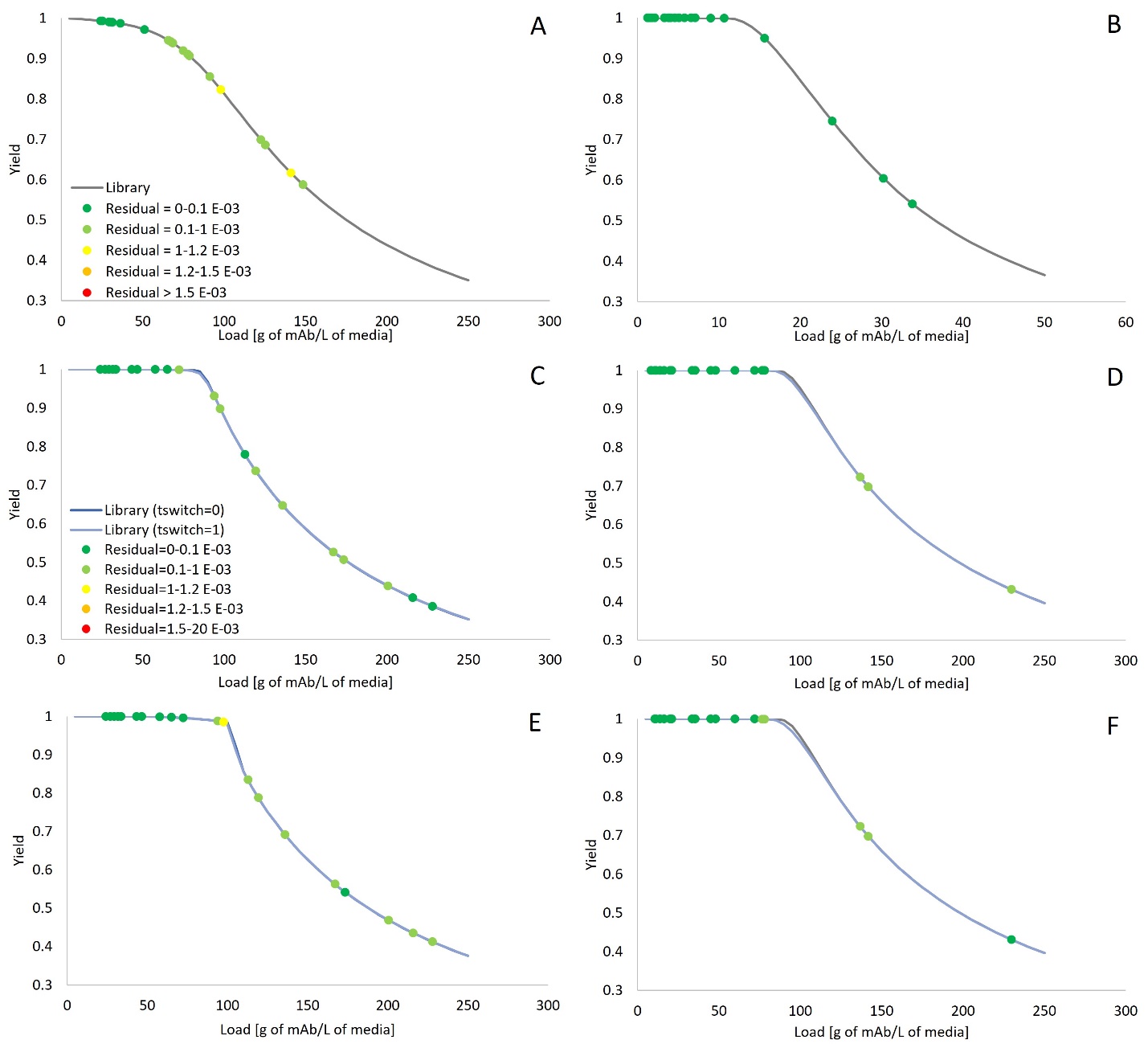


**Figure S5.** Surrogate functions and validation points for describing yield versus relative load for six process platforms: A. Membrane Batch (RMSE = 4.8×10^-4^), B. Resin Batch (RMSE = 8.8×10^-6^), C. Membrane SMB (RMSE = 2.8×10^-4^), D. Resin SMB (RMSE = 7.3×10^-5^), E. Membrane PCC (RMSE = 3.7×10^-4^), and F. Resin PCC (RMSE = 9.4×10^-5^). For MCC platforms requiring the simulation of multiple cycles of load and wash steps, using surrogate functions instead of solving a system of PDEs reduced computing time by two orders of magnitude.

**Table S6.** Normalized minimum lambda value for zero-valued coefficients in LASSO regression for different platforms and KPI. A lower lambda value (red versus blue) indicates a lesser influence of the variable on the KPI. We discarded variables when the normalized lambda values for the two KPIs being optimized were below 0.1.

|  |  | **Membrane** | | | **Resin** | | |
| --- | --- | --- | --- | --- | --- | --- | --- |
|  | Variable | Batch | SMB | PCC | Batch | SMB | PCC |
| Yield | V_col_ | 1.00 | 1.00 | 1.00 | 1.00 | 1.00 | 1.00 |
|  | N_cycle_ | 0.91 | 0.83 | 1.00 | 0.48 | 0.57 | 0.69 |
|  | N_col_ |  | 0.36 | 0.39 |  | 0.25 | 0.25 |
|  | t_switch_ |  | 0.03 | 0.07 |  | 0.02 | 0.02 |
| CU | V_col_ | 1.00 | 1.00 | 1.00 | 1.00 | 1.00 | 1.00 |
|  | N_cycle_ | 1.00 | 0.83 | 0.91 | 0.57 | 0.63 | 0.69 |
|  | N_col_ |  | 0.33 | 0.33 |  | 0.25 | 0.25 |
|  | t_switch_ |  | 0.03 | 0.01 |  | 0.03 | 0.03 |
| Productivity | V_col_ | 1.00 | 0.63 | 0.43 | 1.00 | 1.00 | 1.00 |
|  | N_cycle_ | 0.19 | 0.13 | 0.23 | 0.48 | 0.76 | 0.91 |
|  | N_col_ |  | 1.00 | 1.00 |  | 1.00 | 0.83 |
|  | t_switch_ |  | 0.06 | 0.04 |  | 0.02 | 0.14 |
| COG | V_col_ | 1.00 | 1.00 | 1.00 | 1.00 | 1.00 | 1.00 |
|  | N_cycle_ | 0.91 | 0.76 | 0.83 | 0.76 | 0.76 | 0.83 |
|  | N_col_ |  | 0.33 | 0.33 |  | 0.27 | 0.25 |
|  | t_switch_ |  | 0.00 | 0.01 |  | 0.04 | 0.01 |
| NPV | V_col_ | 1.00 | 1.00 | 1.00 | 1.00 | 1.00 | 1.00 |
|  | N_cycle_ | 0.91 | 0.76 | 0.83 | 0.76 | 0.76 | 0.83 |
|  | N_col_ |  | 0.33 | 0.33 |  | 0.27 | 0.25 |
|  | t_switch_ |  | 0.00 | 0.01 |  | 0.04 | 0.01 |
| PMI | V_col_ | 1.00 | 1.00 | 1.00 | 1.00 | 0.76 | 0.91 |
|  | N_cycle_ | 0.91 | 0.83 | 1.00 | 1.00 | 1.00 | 1.00 |
|  | N_col_ |  | 0.36 | 0.43 |  | 0.27 | 0.52 |
|  | t_switch_ |  | 0.04 | 0.08 |  | 0.03 | 0.25 |
| Prod. Rate | V_col_ | 1.00 | 1.00 | 1.00 | 1.00 | 1.00 | 0.91 |
|  | N_cycle_ | 1.00 | 0.63 | 0.69 | 0.69 | 0.57 | 1.00 |
|  | N_col_ |  | 0.25 | 0.25 |  | 0.25 | 0.27 |
|  | t_switch_ |  | 0.01 | 0.04 |  | 0.04 | 0.09 |

**Table S7.** Variables used for the formulation of the bi-objective optimization problem. Entries marked ‘yes’ indicate that the variable was determined from Lasso regression to be important to the KPIs and was included in the optimization problem. Entries marked ‘no’ indicate that the variable was determined from Lasso regression to be unimportant to the KPIs and was discarded from the optimization problem. The use of LASSO regression reduced the number of variables in 10 of the 18 bi-objective optimization problems we analyzed. It decreased the total number of simulations required by one-third.

|  |  |  | Membrane | | | Resin | | |
| --- | --- | --- | --- | --- | --- | --- | --- | --- |
| Objective 1 | Objective 2 | Variable | Batch | SMB | PCC | Batch | SMB | PCC |
| Maximize Productivity | Maximize CU | V_col_ | Yes | Yes | Yes | Yes | Yes | Yes |
|  |  | N_cycle_ | Yes | Yes | Yes | Yes | Yes | Yes |
|  |  | N_col_ |  | Yes | Yes |  | Yes | Yes |
|  |  | t_switch_ |  | No | No |  | No | Yes |
| Maximize Production  rate | Minimize COG | V_col_ | Yes | Yes | Yes | Yes | Yes | Yes |
|  |  | N_cycle_ | Yes | Yes | Yes | Yes | Yes | Yes |
|  |  | N_col_ |  | Yes | Yes |  | Yes | Yes |
|  |  | t_switch_ |  | No | No |  | No | No |
| Maximize NPV | Minimize PMI | V_col_ | Yes | Yes | Yes | Yes | Yes | Yes |
|  |  | N_cycle_ | Yes | Yes | Yes | Yes | Yes | Yes |
|  |  | N_col_ |  | Yes | Yes |  | Yes | Yes |
|  |  | t_switch_ |  | No | No |  | No | Yes |

**Table S8.** General impacts of increasing independent variables on the KPI.

|  | Membrane KPI | | | | | | |
| --- | --- | --- | --- | --- | --- | --- | --- |
| Variable ↑ | CU | Yield | COG | Productivity | Production Rate | NPV | PMI |
| V_col_ | ↓* | ↑ | ↑ | ≈ | ↑ | ↓ | ↓ |
| N_cycle_ | ↓ | ↑ | ↑ | ≈ | ≈ | ↓ | ↓ |
| N_col_ | ↑ | ≈ | ≈ | ↓ | ↓ | ≈ | ≈ |
| t_switch_ | ↑ | ↓ | ↑ | ≈ | ↑ | ↓ | ≈ |
|  | Resin KPI | | | | | | |
| Variable ↑ | CU | Yield | COG | Productivity | Production Rate | NPV | PMI |
| V_col_ | ↓ | ↑ | ↑ | ↓** | ≈ | ↓ | ↓ |
| N_cycle_ | ↓ | ↑ | ↑ | ↓ | ↓ | ↓ | ↓ |
| N_col_ | ↑ | ≈ | ≈ | ≈ | ≈ | ≈ | ≈ |
| t_switch_ | ↑ | ↓ | ↑ | ≈ | ↑ | ↓ | ≈ |

Note: ↑ increases, ↓ decreases, ≈ little effect

*E.g. The arrow in this cell indicates that as column volume (V_col_) increases, capacity utilization (CU) decreases. Although the larger volume allows for capturing more material, the column becomes oversized when all other process conditions remain unchanged, resulting in underutilized capacity (reflected by a lower CU).

** The red symbols indicate that they differ from the corresponding symbols for membranes.
